# Endurance training lowers ribosome density despite increasing ribosome biogenesis markers in rodent skeletal muscle

**DOI:** 10.1186/s13104-017-2736-0

**Published:** 2017-08-11

**Authors:** Matthew A. Romero, C. Brooks Mobley, Melissa A. Linden, Grace Margaret-Eleanor Meers, Jeffrey S. Martin, Kaelin C. Young, R. Scott Rector, Michael D. Roberts

**Affiliations:** 1 School of Kinesiology, Auburn University, Auburn, AL USA; 2 Department of Cell Biology and Physiology, Edward Via College of Osteopathic Medicine-Auburn Campus, Auburn, AL USA; 3 Medicine-Division of Gastroenterology and Hepatology, and Nutrition and Exercise Physiology, University of Missouri, Columbia, MO USA; 4 Research Service, Harry S Truman Memorial VA Hospital, Columbia, MO USA; 5 School of Kinesiology, Molecular and Applied Sciences Laboratory, Edward Via College of Osteopathic Medicine-Auburn Campus, Auburn University, 301 Wire Road, Office 286, Auburn, AL 36849 USA

**Keywords:** Ribosome biogenesis, Endurance training, Western diet, Ribophagy, rRNA degradation

## Abstract

**Objective:**

The purpose of this study was to examine if: (a) high sugar/high fat Western diet (WD)-feeding affects skeletal muscle ribosome biogenesis markers in hyperphagic, diabetic-prone Otsuka Long-Evans Tokushima Fatty (OLETF) rats, and (b) 12 weeks of treadmill training rescued potential detriments that WD feeding exerted on these markers.

**Methods:**

Eight week-old male OLETF rats were fed a low-fat control diet (O-CON, n = 10) or high/sucrose/cholesterol Western diet (WD). At weeks 20–32 of age, WD-fed rats were divided into WD sedentary (O-WD/SED, n = 16), or WD treadmill trained (5 days/week, 60 min/day) (O-WD/EX, n = 10) conditions.

**Results:**

Interestingly, total RNA (i.e., ribosome density) was 2.3-fold greater in O-WD/SED versus O-WD/EX rats (p = 0.003) despite levels of upstream binding factor protein, RNA polymerase I protein and pre-45S rRNA being greater in O-WD/EX rats. Ribophagy (USP10 and G3BP1) and TRAMP-exosome rRNA degradation pathway (EXOSC10 and SKIV2L2) proteins were assayed to determine if these pathways were involved with lower ribosome density in O-WD/EX rats. While USP10 was higher in O-CON versus O-WD/SED and O-WD/EX rats (p < 0.001 and p < 0.001, respectively), G3BP1, EXOSC10 and SKIV2L2 did not differ between groups. Nop56 and Ncl mRNAs, ribosome assembly markers, were highest in O-WD/EX rats. However, Fbl mRNA and 28S rRNA, downstream ribosome processing markers, were lowest in O-WD/EX rats. Collectively these data suggest that, in WD-fed rats, endurance training increases select skeletal muscle ribosome biogenesis markers. However, endurance training may reduce muscle ribosome density by interfering with rRNA processing and/or export through mechanisms independent of ribophagy or rRNA degradation.

## Introduction

Type II diabetes (T2D) impairs anabolic signaling in skeletal muscle as shown by Stephens et al. [1] who reported a decrease in eukaryotic initiation factor 4E binding protein 1 (4EBP1) phosphorylation which, in turn, was associated with decrements in overall muscle protein synthesis. Ribosome biogenesis is a critical regulator of muscle mass given that it catalyzes muscle protein synthesis, and there has been an increased emphasis on investigating the mechanisms that regulate this process [2]. Acute and chronic resistance exercise increases markers of ribosome biogenesis [3, 4]. However, no research has examined how chronic endurance exercise affects the process of ribosome biogenesis. Endurance training improves other facets of skeletal muscle physiology in diabetic rodents (i.e., insulin signaling, mitochondrial biogenesis and microvascular physiology) [5–7]. Thus, it stands to reason that endurance training may also improve markers of ribosome biogenesis in diabetic rats which, in turn, facilitates other improvements in skeletal muscle metabolism. Thus, the purpose of this study was to examine if: (a) high sugar/fat/cholesterol (i.e., Western diet) feeding affects various markers of skeletal muscle ribosome biogenesis in hyperphagic, diabetic-prone Otsuka Long-Evans Tokushima Fatty (OLETF) compared to OLETF rats fed a low-fat control diet, and (b) if 12 weeks of endurance training rescued potential detrimental effects that Western diet feeding exerted on skeletal muscle ribosome biogenesis markers (i.e., comparing sedentary and endurance-trained rats fed a Western diet).

## Main text

### Methods

#### Animal protocol

The animal protocol was approved by the Institutional Animal Care and Use Committee at the University of Missouri and complied with the National Institutes of Health’s Guide for the Care and Use of Laboratory Animals. Muscle samples assayed in the current study were obtained from male OLETF rats (Tokushima Research Institute, Otsuka Pharmaceutical, Tokushima, Japan) from a prior investigation [8]. Notably, OLETF rats are hyperphagic and become obese hyperglycemic after 18 weeks of age [9]. Briefly, 8-week old OLETF rats were randomized to either consume a control diet (O-CON, n = 10) (Diet D12110704, Research Diets Inc.; 10% kcal fat, 70% kcal carbohydrate and 20% kcal protein, with 3.5% kcal sucrose) or Western diet (WD, n = 26) (Diet D09071604, Research Diets Inc.; 44.9% kcal fat, 35.1% kcal carbohydrate and 20% kcal protein, with 1% weight/weight from cholesterol and 17% kcal sucrose) for 24 weeks (up to 32 weeks of age). At 20 weeks of age, the O-WD animals were assigned one of two subset groups which included a sedentary (O-WD/SED, n = 16) or exercise trained (O-WD/EX, n = 10) condition for 12 weeks. Throughout the protocol, all animals were individually housed in standard conditions (0600/1800 h light/dark cycle at 21 °C) and diet and water were provided ad libitum. At 32 weeks of age, animals were overnight fasted and, the morning of dissection, were anaesthetized by intraperitoneal injection of sodium pentobarbital (100 mg/kg) and were euthanized thereafter by exsanguination. *Vastus lateralis* muscles were obtained via standard dissection technique, flash frozen in liquid nitrogen and stored at −80 °C until analyses described below.

#### Exercise training for O-WD/EX rats

At 20 weeks of age, O-WD/EX began treadmill running 5 days/week as described previously [8]. The speed and duration of the treadmill exercise were gradually increased over the first 4 weeks of training until the animals could maintain a running speed of 20 m/min for 60 min/day. By the fifth week of training, animals ran at 20 m/min, 60 min/day, on a 15% incline and maintained this until 32 weeks of age. Animals in the O-SED were placed on a non-moving treadmill weekly.

#### Western blotting procedures

In-depth Western blotting procedures are similar to what our laboratory have previously published [3, 10]. Notably, primary antibodies used included the following: (1) rabbit anti-rat RNA polymerase I (RNA Pol I) (1:1000; Thermo Scientific, Rockford, IL, USA), (2) mouse anti-rat upstream binding factor (UBF) (1:1000; Santa Cruz Biotechnology, Dallas, TX, USA), (3) rabbit anti-rat c-myelocytomatosis oncogene (c-Myc) (1:1000; Cell Signaling, Danvers, MA, USA), (4) ubiquitin-specific peptidase 10 (USP10) (1:1000, Cell Signaling), (5) GTPase activating protein binding protein 1 (G3BP1) (1:1000; Santa Cruz Biotechnology, Dallas, TX, USA), (6) mouse anti-rat exosome component 10 (EXOSC10) (1:1000; Santa Cruz Biotechnology, Dallas, TX, USA), (7) mouse anti-rat Superkiller Viralicidic Activity 2-Like 2 (SKIV2L2) (1:1000; Santa Cruz Biotechnology, Dallas, TX, USA).

#### Total RNA determination and real-time PCR

In-depth total RNA isolation/quantification and real-time PCR methods utilized are similar to what our laboratory have previously published [3, 10], and details regarding PCR primers as well as fold-change calculations have been previously published [3]. Of note, cyclophilin A was used as a housekeeping gene for fold-change calculations given that it remained stable across all diet and activity treatments.

#### Subcellular protein determination

In-depth protein isolation/quantification methods utilized are similar to what our laboratory have previously published [10]. Due to limited tissue, only a subset of animals were able to be assayed per group (O-CON n = 7, O-WD/SED n = 14, O-WD/EX n = 8).

#### Statistical analyses

All data are presented in figures as mean ± standard deviation (SD) values. Statistics were performed using SPSS v22.0 (IBM, Armonk, NY, USA). All dependent variables were compared between treatments using one-way ANOVAs with post hoc independent t test with Bonferroni correction being performed when ANOVA p values were <0.05. The partial eta squared statistic (ɳ^2^) was calculated for effect size for all ANOVAs, and values between 0.010 and 0.059, values between 0.060 and 0.138 and values greater than 0.138 can be interpreted as small, medium, and large effect sizes, respectively. Likewise, 95% confidence intervals are presented for all dependent variables.

### Results

Body mass, food consumption, serum glucose, serum insulin and homeostatic model assessment of insulin resistance (HOMA-IR) values from each group are presented in Table 1 with accompanying ANOVA p values, effect sizes, and 95% confidence intervals. Note that these values are partial n-sizes of data previously presented by Linden et al. [8], and serve to provide information regarding the phenotype of each group for convenience to the reader. Body mass was greater in O-WD/SED versus O-CON (p < 0.05), caloric consumption during weeks 20–32 was greater in O-WD/SED versus other groups (p < 0.05), and fasted serum glucose was lowest in O-WD/EX versus other groups (p < 0.05). HOMA-IR values were significantly greater in O-CON versus O-WD/EX rats (p = 0.021), suggesting that O-CON rats presented a worsened degree of insulin resistance compared to O-WD/SED rats. While HOMA-IR values were 67% greater in O-WD/SED versus O-WD/EX rats, this failed to reach statistical significance (p = 0.123).

**Table 1:** Body mass, food consumption and serum glucose and insulin values

Variable	Values (mean ± SD)	95% CI	ANOVA p value (eta-squared)
Sacrifice body mass (g)			
O-CON	648 ± 80^a^	594–718	0.032
O-WD/SED	726 ± 87^b^	686–787	(0.188)
O-WD/EX	720 ± 36^a,b^	696–744	
Kcal consumed (week 20–32)			
O-CON	746 ± 56^a^	682–768	<0.001
O-WD/SED	843 ± 39^b^	803–848	(0.535)
O-WD/EX	758 ± 31^a^	736–780	
Glucose (mg/dL)			
O-CON	339 ± 40^a^	306–371	0.001
O-WD/SED	341 ± 65^a^	303–338	(0.364)
O-WD/EX	253 ± 44^b^	222–284	
Insulin (ng/dL)			
O-CON	5.1 ± 3.2	2.9–7.9	0.11
O-WD/SED	3.4 ± 2.6	2.2–5.2	(0.124)
O-WD/EX	2.7 ± 1.2	1.9–3.6	
HOMA-IR			
O-CON	4.4 ± 3.1^a^	2.3–7.1	0.023
O-WD/SED	2.8 ± 2.1^a,b^	1.7–4.2	(0.205)
O-WD/EX	1.7 ± 0.7^b^	1.2–2.1	

These values are partial n-sizes of data previously presented by Linden et al. [15], and serve to provide information regarding the phenotype of each group for convenience to the reader. All data are presented as mean ± SD (n = 10–16 rats per group), 95% confidence intervals (CI) are presented, and data with different superscript letters indicate between-treatment differences (p < 0.05)

*O-CON* OLETF rats consuming a control diet during weeks 20–32 and were not treadmill-trained, *O-WD/SED* OLETF rats consuming a Western diet during weeks 20–32 and were not treadmill-trained, *O-WD/EX* OLETF rats consuming a Western diet during weeks 20–32 and were treadmill-trained 5 days/week; HOMA-IR, homeostatic model assessment of insulin resistance

**Fig. 1:**
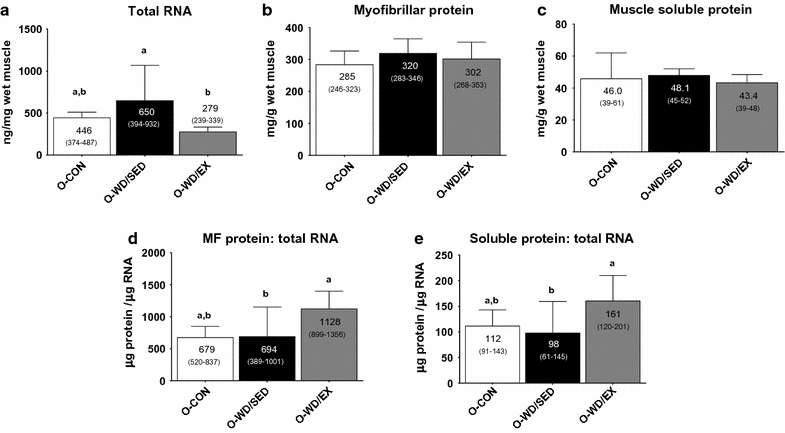
Effects of diet and exercise on markers of total ribosome content and function. The effects of the intervention on skeletal muscle total RNA levels (i.e., ribosome content) (**a**), myofibrillar protein levels (**b**), muscle soluble protein levels (**c**), myofibrillar (MF): total RNA ratio as a surrogate of ribosome function (**d**) and muscle soluble protein: total RNA ratio as an additional surrogate of ribosome function (**e**). All data are presented as mean ± SD (n = 10–16 rats per group), group mean values are presented within each bar, 95% confidence intervals are presented in *parentheses within each bar*, and *bars with different superscript letters* indicate between-treatment differences (p < 0.05). Due to limited tissue, only a subset of animals were assayed for myofibrillar protein (O-CON n = 7, O-WD/SED n = 14, O-WD/EX n = 8). *O-CON* OLETF rats consuming a control diet during weeks 20–32 and were not treadmill-trained, *O-WD/SED* OLETF rats consuming a Western diet during weeks 20–32 and were not treadmill-trained, *O-WD/EX* OLETF rats consuming a Western diet during weeks 20–32 and were treadmill-trained 5 days/week

**Fig. 2:**
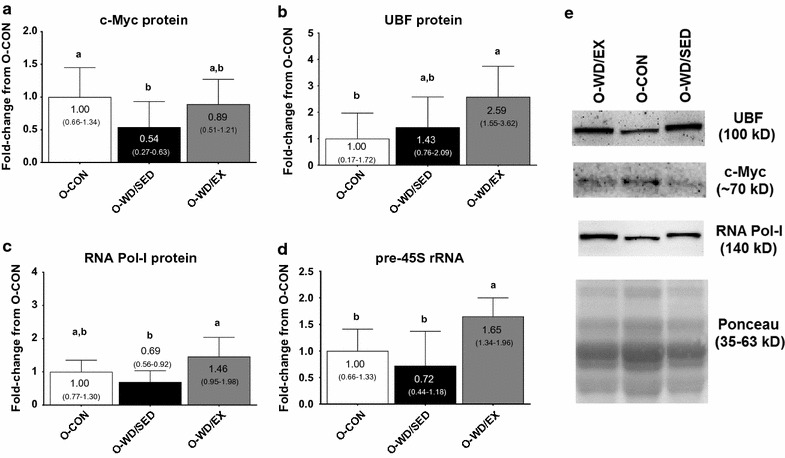
Effects of diet and exercise on markers of ribosome biogenesis. The effects of the intervention on c-Myc protein levels (**a**), UBF protein levels (**b**), RNA Pol I protein levels (**c**) and pre-45S rRNA transcript levels (**d**). **e** Representative images of Western blotting targets. All data are presented as mean ± SD (n = 10–16 rats per group), group mean values are presented within each bar, 95% confidence intervals are presented in *parentheses within each bar*, and *bars with different superscript letters* indicate between-treatment differences (p < 0.05). *O-CON* OLETF rats consuming a control diet during weeks 20–32 and were not treadmill-trained, *O-WD/SED* OLETF rats consuming a Western diet during weeks 20–32 and were not treadmill-trained, *O-WD/EX* OLETF rats consuming a Western diet during weeks 20–32 and were treadmill-trained 5 days/week

Total RNA (ANOVA p = 0.010, ɳ^2^ = 0.260) was significantly greater in the O-WD/SED than the O-WD/EX rats (p = 0.003) (Fig. 1a). Myofibrillar protein (ANOVA p = 0.260, ɳ^2^ = 0.092; Fig. 1b) and muscle soluble protein (ANOVA p = 0.547, ɳ^2^ = 0.040; Fig. 1c) was not different between groups. The ratio of myofibrillar protein to total RNA (ANOVA p = 0.024, ɳ^2^ = 0.278), a gross marker of translational efficiency [11], was significantly greater in O-WD/EX versus O-WD/SED rats (p = 0.040), and tended to be greater in O-WD/EX versus O-CON rats (p = 0.061) (Fig. 1d). The ratio of muscle soluble protein to total RNA (ANOVA p = 0.032, ɳ^2^ = 0.226), another gross marker of translational efficiency [11, 12], was significantly greater in O-WD/EX compared to O-WD/SED (p = 0.031), but was not different between O-CON and O-WD/EX rats (p = 0.167) (Fig. 1e).

c-Myc protein levels (ANOVA p = 0.019, ɳ^2^ = 0.219) were significantly greater in O-CON compared to O-WD/SED (p = 0.030), but not O-WD/EX rats (Fig. 2a). UBF protein levels (ANOVA p = 0.011, ɳ^2^ = 0.259) were significantly greater in O-WD/EX versus O-CON rats (p = 0.012), but not different from O-WD/SED rats (Fig. 2b). RNA Pol I protein levels (ANOVA p < 0.001, ɳ^2^ = 0.383) were significantly greater in O-WD/EX versus O-WD/SED rats (p < 0.001), and tended to be higher in the former group versus O-CON rats (p = 0.061) (Fig. 2c). Pre-45S rRNA (ANOVA p = 0.001, ɳ^2^ = 0.360) was significantly greater in O-WD/EX versus O-WD/SED and O-CON rats (p = 0.001 and p = 0.034, respectively) (Fig. 2d).

**Fig. 3:**
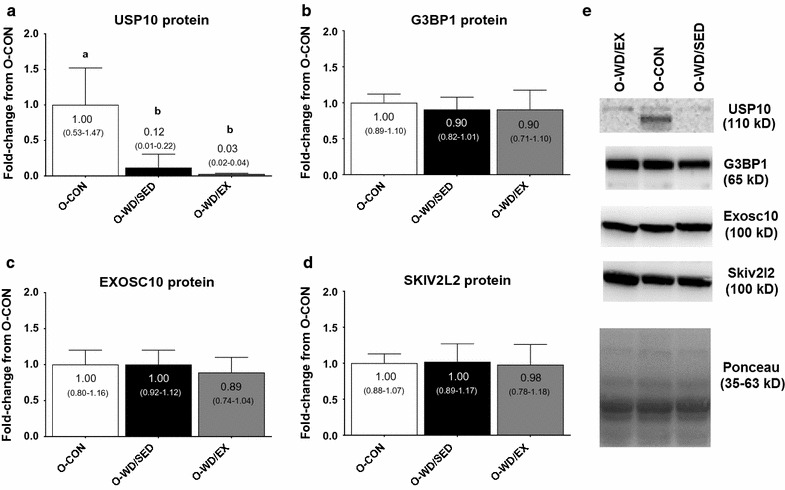
Effects of diet and exercise on markers of ribosome degradation. The effects of the intervention on USP10 protein levels (**a**) and G3BP1 protein levels (**b**) (both markers being critical for ribophagy), as well as EXOSC10 protein levels (**c**) and SKIV2L2 protein levels (**d**) (both markers being the TRAMP exosome rRNA degradation pathway). **e** Representative images of Western blotting targets. All data are presented as mean ± SD (n = 10–16 rats per group), group mean values are presented within each bar, 95% confidence intervals are presented in *parentheses within each bar*, and *bars with different superscript letters* indicate between-treatment differences (p < 0.05). *O-CON* OLETF rats consuming a control diet during weeks 20–32 and were not treadmill-trained, *O-WD/SED* OLETF rats consuming a Western diet during weeks 20–32 and were not treadmill-trained, *O-WD/EX* OLETF rats consuming a Western diet during weeks 20–32 and were treadmill-trained 5 days/week

USP10 protein levels (ANOVA p < 0.001, ɳ^2^ = 0.675) were significantly greater in O-CON versus O-WD/SED and O-WD/EX rats (p < 0.001 and p < 0.001, respectively) (Fig. 3a), while protein levels of G3BP1 (ANOVA p = 0.431, ɳ^2^ = 0.508; Fig. 3b) EXOSC10 (ANOVA p = 0.385, ɳ^2^ = 0.056; Fig. 3c) or SKIV2L2 (ANOVA p = 0.908, ɳ^2^ = 0.006; Fig. 3d) were not different between groups.

**Fig. 4:**
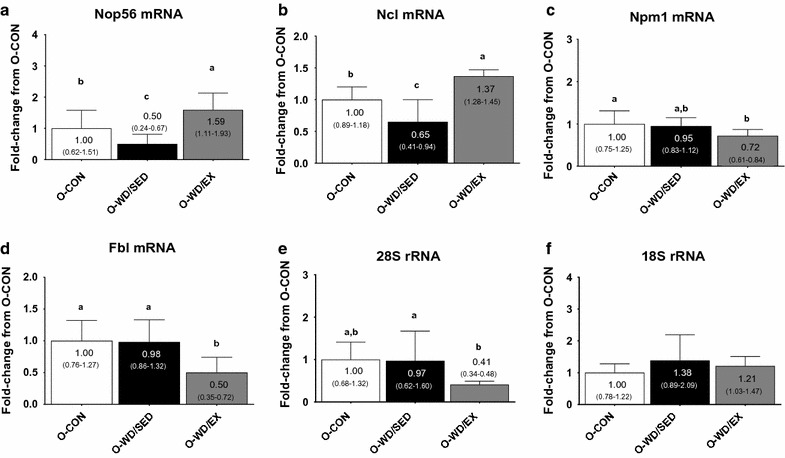
Effects of diet and exercise on the expression of genes associated with rRNA processing and ribosome export. The effects of the intervention on Nop56 mRNA levels (**a**) and Ncl mRNA levels (**b**) (both genes being involved in rRNA processing and ribosome assembly), Npm1 mRNA levels (**c**) and Fbl mRNA levels (**d**) (genes involved with nuclear ribosome export), and 28S rRNA transcript levels (**e**) and 18S rRNA transcript levels (**f**) (both markers being indicative of downstream rRNA processing from the pre-45S transcript). All data are presented as mean ± SD (n = 10–16 rats per group), group mean values are presented within each bar, 95% confidence intervals are presented in *parentheses within each bar*, and *bars with different superscript letters* indicate between-treatment differences (p < 0.05). *O-CON* OLETF rats consuming a control diet during weeks 20–32 and were not treadmill-trained, *O-WD/SED* OLETF rats consuming a Western diet during weeks 20–32 and were not treadmill-trained, *O-WD/EX* OLETF rats consuming a Western diet during weeks 20–32 and were treadmill-trained 5 days/week

Nop56 mRNA (ANOVA p < 0.001, ɳ^2^ = 0.508) was significantly greater in O-WD/EX versus O-CON and O-WD/SED rats (p = 0.024 and p < 0.001, respectively), as well as O-CON versus O-WD/SED rats (p = 0.034) (Fig. 4a). Ncl mRNA (ANOVA p < 0.001, ɳ^2^ = 0.588) was significantly greater in O-WD/EX versus O-CON and O-WD/SED rats (p = 0.011 and p < 0.001, respectively), as well as O-CON versus O-WD/SED rats (p = 0.009) (Fig. 4b). Npm1 mRNA (ANOVA p = 0.024, ɳ^2^ = 0.216) was significantly greater in O-CON versus O-WD/EX rats (p = 0.033) (Fig. 4c). Fbl mRNA (ANOVA p = 0.001, ɳ^2^ = 0.347) was significantly greater in O-CON (p = 0.002) and O-WD/SED (p = 0.004) versus O-WD/EX rats (Fig. 4d). 28S rRNA (ANOVA p = 0.023, ɳ^2^ = 0.223) was significantly lower in O-WD/EX versus O-WD/SED rats (p = 0.04) (Fig. 4e). There were no between-group differences in 18S rRNA (ANOVA p = 0.316, ɳ^2^ = 0.074).

## Discussion

Two prior studies [13, 14] have reported that skeletal muscle ribosome density was lower in diabetic animals compared to control animals, suggesting that the severity of diabetes and/or insulin resistance may be associated with reduced ribosome density. However, we observed a low ribosome density (total RNA) in O-WD/EX rats despite these rats presenting significantly lower HOMA-IR scores compared to the O-CON rats and lower (albeit non-significant) HOMA-IR scores compared to O-WD/SED rats (Table 1). Therefore, these results are suggestive of an endurance exercise-induced decrease in ribosome density in endurance-trained OLETF rats despite a lower severity of insulin resistance compared to sedentary control and WD-fed rats.

Although investigations have reported that skeletal muscle ribosome function (i.e., muscle protein synthesis) increases acutely with endurance exercise [15, 16], no one has examined whether chronic endurance training affects ribosome content and/or markers of ribosome biogenesis and turnover in skeletal muscle. Compared to one or both of the sedentary rat groups (O-CON and O-WD/SED), O-WD/EX rats presented with: (a) the greatest levels of UBF protein, RNA Pol I protein and pre-45S rRNA which is suggestive of increased ribosome biogenesis, (b) the lowest levels of total RNA, and (c) higher expression of select mRNAs related to ribosome processing and formation (i.e., Ncl and Nop56).

We next assayed ribophagy and TRAMP-exosome pathway targets given that ribosome density was lower in endurance exercised rats. Ribophagy is a selective process in which the autophagy machinery selectively targets mature ribosomes for degradation [17, 18], and involves a protease complex that consists of USP10 and its associated protein G3BP1. The complex acts to de-ubiquitylate the 60S ribosomal subunit and signals the autophagasome to engulf the subunit for degradation [18]. The TRAMP-exosome pathway involves rRNA degradation through intracellular exosomes whereby rRNA is degraded via the exosome’s 3′→5′ exonuclease activity [19]. Interestingly, skeletal muscle USP10 protein robustly decreased in both the O-WD/SED and O-WD/EX compared to O-CON rats suggesting that WD feeding downregulates the expression of this ribophagy enzyme; notably, neither G3BP1 nor TRAMP exosome pathway markers were significantly different between groups. We next interrogated the mRNA expression of genes related to rRNA processing (Nop56), ribosome assembly (Ncl), ribosome processing from the pre-45S rRNA transcript (28S rRNA and 18S rRNA) and nuclear ribosome export (Npm1 and Fbl) in order to examine if these targets were lower in exercised rats. Similar to UBF protein, RNA pol I protein and pre-45S rRNA expression patterns, the expression of select genes involved with rRNA processing and ribosome assembly (Nop56 and Ncl) were greatest in exercised rats. Hence, in the WD rats studied herein, endurance training increases certain markers of ribosome biogenesis as well as certain mRNAs related to downstream ribosome processing. However, Npm1 and Fbl mRNA (genes related to nuclear ribosome export) and 28S rRNA levels (indicative of rRNA processing from the pre-45S transcript) were lowest in the O-WD/EX group. Therefore, endurance exercise may interfere with rRNA processing and ribosome export through an unidentified mechanism that was not related to ribosome turnover via ribophagy and/or the TRAMP-exosome pathway.

While non-exercised rats exhibited a greater skeletal muscle ribosome density, gross markers of ribosome function were greater in endurance-trained rats. In this regard, O-WD/EX rats presented with the greatest levels of myofibrillar protein to RNA and soluble muscle protein to RNA ratios which is suggestive of increased ribosome function; this finding being in accordance reports suggesting that endurance exercise increases myofibril and mitochondrial protein synthesis rates in rodents [20]. Thus, while endurance exercise may reduce ribosome formation, it does not seem to impair ribosome function in OLETF rats given that myofibril and total soluble muscle protein was not reduced with endurance training.

## Limitations

First, only one sacrificial time point was assessed which precludes us from determining time course mechanisms as to how the assayed markers were altered throughout the intervention. Second, we did not include a non-OLETF control strain in the current study (i.e., leaner LETO rats), so comparing the assayed markers between diabetic-prone OLETF and LETO rats in future studies would yield intuitive information regarding how ribosome biogenesis is affected between strains. Likewise, we did not include an O-CON/EX group which could have provided more in-depth information regarding how both diets with and without exercise affected the markers assayed herein. Notwithstanding, these data provide novel evidence examining how chronic endurance exercise affects processes related to skeletal muscle ribosome dynamics, and therefore, warrants future work in humans to confirm these findings.

## Abbreviations

4EBP1: eukaryotic initiation factor 4E binding protein 1
ANOVA: analysis of variance
c-Myc: c-myelocytomatosis oncogene
EX: exercise trained
EXOSC10: exosome component 10
Fbl: fibrillarin
G3BP1: GTPase activating protein binding protein 1
HKG: housekeeping gene
HOMA-IR: homeostatic model assessment of insulin resistance
Ncl: nucleolin
Npm1: nucleophosmin
OLETF: Otsuka Long-Evans Tokushima Fatty
PolI/III: RNA polymerase I/III
rRNA: ribosomal ribonucleic acid
SED: sedentary
SKIV2L2: Superkiller Viralicidic Activity 2-Like 2
T2D: type II diabetes
UBF: upstream binding factor
USP10: ubiquitin-specific peptidase 10
WD: Western diet
